# Bridging heart failure clinic–based guideline-directed medical therapy optimization and real-world practice in heart failure with reduced ejection fraction (HFrEF)

**DOI:** 10.1093/eschf/xvag078

**Published:** 2026-03-13

**Authors:** Taner Sen, Mevlut Demir, Ceyda Nur Batak

**Affiliations:** Cardiology Department, Kutahya Health Sciences University, Kutahya, Türkiye; Cardiology Department, Kutahya Health Sciences University, Kutahya, Türkiye; Cardiology Department, Kutahya Health Sciences University, Kutahya, Türkiye

**Keywords:** Heart failure with reduced ejection fraction (HFrEF), Guideline adherence, Real-world practice, Türkiye

We read with great interest the nationwide analysis by Berge *et al*. entitled ‘Improvements in medical therapy and prognosis for patients with heart failure with reduced ejection fraction (HFrEF) following the 2021 ESC HF guidelines’ demonstrating a marked increase in guideline-directed medical therapy (GDMT) utilization and a parallel reduction in heart failure hospitalizations following the 2021 ESC heart failure guidelines. The rapid uptake of mineralocorticoid receptor antagonists and sodium–glucose cotransporter-2 inhibitors, together with the high proportion of patients achieving ≥50% of target doses, represents an outstanding example of successful guideline implementation at a national level.^1^ An important strength of this study is that it reflects care delivered within structured heart failure outpatient clinics with systematic uptitration protocols and dedicated multidisciplinary follow-up. This highly organized infrastructure likely played a central role in achieving the impressive GDMT optimization rates reported.

In this context, our contemporary recently published nationwide HF-PERSPECTIVE survey entitled ‘Clinical Approaches in Heart Failure: Perspectives of Cardiologists in Türkiye (HF-PERSPECTIVE)’ enrolled 495 cardiologists in Türkiye providing high-resolution insight into practice heterogeneity within a uniform healthcare system.^2^ In addition, the more recent conduct of our study allows an assessment of how the latest guideline updates have influenced real-world clinical practice. The sequencing patterns observed in HF-PERSPECTIVE indicate persistent reliance on the traditional stepwise approach, with therapy typically initiated with an angiotensin-converting enzyme inhibitor (ACEi) or an angiotensin receptor–neprilysin inhibitor (ARNI), followed by beta-blockers (BBs), mineralocorticoid receptor antagonists (MRAs), and sodium–glucose cotransporter 2 inhibitors (SGLT2is). Achievement of target or maximally tolerated doses remained suboptimal: 45.5% of respondents reached these doses in only 30%–50% of their patients and 30.5% in fewer than 30%. Moreover, reported timelines for dose optimization suggested delayed achievement of target therapy doses. Nearly half of the respondents (48.5%) reported requiring 1–3 months to reach target/maximally tolerated doses of the 4 foundational therapies, and 27.9% reported 3–6 months, whereas only 14.7% reported achieving optimization within 1 month.

Importantly, our study addressed several practice-related questions. In a hypothetical single-drug scenario, when asked to choose a single therapy if only one drug was available for HFrEF, 64% selected ARNI, followed by SGLT2i (18.2%), BBs (12.5%), and MRAs (5.3%). In acute *de novo* HF, 63% favoured initiating an ARNI over an ACE inhibitor when feasible, whereas this preference declined to 45.1% in the outpatient setting; 46.5% reported switching eligible outpatients from ACEi/ARB to ARNI irrespective of functional class. Initial ARNI dosing was 24/26 mg twice daily in 83.8% of ARNI-naïve patients and 49/51 mg twice daily in 54.7% of those previously receiving ACE inhibitor/ARB. Uptake of SGLT2i in the acute setting was high (94.9%).

Decongestion strategies were heterogeneous, and objective response measures were underused. Only 31.1% assessed diuretic response with urinary sodium, and discharge decisions were guided mainly by clinical signs rather than biomarker changes. Continuous intravenous furosemide infusion was preferred over bolus dosing, and sequential nephron blockade was initiated early in approximately half of the respondents, with oral acetazolamide incorporated by 62.4%.

Taken together, these findings suggest that the improvement in outcomes observed in the present study is not solely a consequence of updated guideline recommendations but also of the healthcare infrastructure capable of delivering rapid, protocol-driven GDMT optimization. In healthcare systems where structured heart failure clinics, close early follow-up, and multidisciplinary pathways are not consistently available, achieving similar treatment intensity remains challenging. We believe that the study by Berge *et al*. provides a model for how national heart failure programmes can successfully translate guideline recommendations into clinical benefit. Future implementation strategies should therefore focus not only on drug adoption but also on the development of dedicated care pathways that enable early and systematic uptitration.

## Data Availability

Data supporting the findings of this study are available from the corresponding author upon reasonable request.
